# Does mental fatigue influence the accuracy of paralympic boccia players?

**DOI:** 10.3389/fspor.2025.1724991

**Published:** 2026-02-03

**Authors:** Diego Francisco da Silva, André dos Santos Costa, Leonardo de Sousa Fortes, Saulo Fernandes de Melo Oliveira

**Affiliations:** 1 Department of Physical Education, Federal University of Pernambuco, Recife, Brazil; 2 Department of Sports, Federal University of Minas Gerais, Belo Horizonte, Brazil

**Keywords:** cognitive effort, cognitive fatigue, mental demand, paralympic sport, sports performance

## Abstract

Mental fatigue (MF) is characterized by a long period of low cognitive complexity or a short period of high cognitive demand, moreover, MF decreases accuracy in the sport. Thus, the objective of this study was to examine the impact of MF on accuracy in Paralympic boccia (PB) athletes. A total of 11 PB athletes participated in the study, including 6 with cerebral palsy (CP) and 5 without CP. Each participant underwent three experimental conditions: MF, low cognitive effort (LCE), and control (C). All procedures were conducted at the athletes' training facilities. Accuracy was assessed following the experimental protocols. A generalized estimating equations (GEE) model was used to analyze the effects of time and condition. A significant time effect was observed for both the LCE (*p* < 0.05) and MF (*p* < 0.05) conditions. A condition effect was found for mental demand (LCE vs. MF; *p* < 0.05). Regarding accuracy, no condition effect was observed for the 3, 6, and 9-m distances or for the total score (*p* > 0.05). In conclusion, the present study demonstrated that LCE and mental fatigue do not negatively affect the accuracy of PB athletes.

## 1 Introduction

The MF is characterized either by a prolonged period of low cognitive complexity or by a short period of high cognitive demand, both of which are associated with a decline in executive function performance, followed by sensations of tiredness and lack of energy (1, 2). Moreover, the relationship between elevated states of MF and the various components of physical performance has been the focus of investigation in sports science in recent years (3–10).

From the perspective of the effects of MF on technical performance in sports, a review with meta-analysis demonstrated that MF significantly impacts both offensive and defensive skills in soccer (11). In addition to negatively affecting the distance covered and the accuracy of passes and shots in soccer players (12). Regarding basketball, MF impairs decision-making and decreases performance in free-throw and three-point shooting techniques (13).

Regarding the impact of MF on accuracy in sports, it has been found that MF reduces the performance of table tennis athletes by decreasing accuracy and ball speed, while increasing the number of errors committed (14). In addition, MF decreases the number of points scored and increases the number of points missed in a shooting task performed by collegiate basketball athletes (4). From this perspective, MF led to an increase in the number of errors, omissions, and response time in a rifle marksmanship test (15).

Given the above, it is evident that the literature has advanced in exploring the impacts of MF on sports performance. However, when it comes to Paralympic sports, the literature on the relationship between MF and these disciplines remains limited. Nonetheless, a recent study found that MF negatively affected the physical performance of wheelchair basketball athletes (16), providing evidence of potential adverse effects on paralympic sports. Based on this, Paralympic sports are practiced by individuals with motor, sensory, and intellectual impairments.

Accordingly, athletes with cerebral palsy (CP) require greater cognitive demands to maintain postural control (17, 18), They also exhibit greater activation in the prefrontal cortex when performing a motor task and poorer performance in executive functions, cognitive flexibility, attentional control, selective attention, and information processing when compared with the general population (19–21). Thus, individuals with CP and brain injury are more likely to experience MF than the general population (22–24).

From this perspective, Boccia is a sport practiced by individuals with CP, tetraplegia, congenital malformations, or severe degenerative diseases. In this context, PB has gained prominence in international competitions within the national Paralympic scene. The PB is a sport that can be practiced by individuals of any gender, either individually, in pairs, or in teams of three. Its practice is closely associated with tactical and technical awareness, high levels of muscular control, concentration, and accuracy. The objective is to position one's ball as close as possible to the target ball on the court through precise throws (25, 26).

It comprises four athlete categories, known as functional classes (FC), which are defined based on the athlete's level of functionality during gameplay (27). Athletes in FC BC1 exhibit difficulties in propelling a manual wheelchair and performing movements, whereas those in FC BC2 have impairments in isolated movements but are able to maneuver the wheelchair and throw the ball without assistance. In BC3, athletes present significant locomotor dysfunction, along with insufficient coordination and strength to handle the ball, requiring assistive devices and support. In FC BC4, athletes demonstrate severe dysfunction but maintain sufficient capacity to play and maneuver the wheelchair independently (28).

Additionally, no studies have been identified in the literature that have examined the effects of protocols designed to induce MF in a sport with high strategic, tactical, and technical demands such as Paralympic boccia. Thus, could prior cognitive effort induce MF in PB athletes and consequently impair accuracy in a specific test? Given this context, it is of utmost importance to examine the impact of MF on the technical performance of Boccia athletes.

## 2 Materials and methods

### 2.1 Participants

The recruitment of PB athletes involves a population of limited size; therefore, no *a priori* analyses were conducted to estimate the sample size. The athletes were selected through a non-probabilistic convenience sampling method. A total of 11 Boccia athletes (6 with CP and 5 without CP) participated in the study, with a mean age of 29.9 ± 10.1 years and an average of 94.4 ± 37.6 months of experience in the sport. Regarding competitive level, seven athletes were classified as level 3 (local, regional, and national), two as level 4 (international and national team), one as level 5 (top 3 in the world ranking), and one as level 2 (regional level), based on the classification of (29).

All participants signed the informed consent form. The present research project has been approved by the research ethics committee under the registry CAAE: 39,603,920.9.0000.9430. All athletes signed the informed consent form.

### 2.2 Experimental design

The research is characterized as experimental of the crossover type (30, 31). Each individual underwent one familiarization session and three experimental conditions: a C session, a LCE session, and a MF induction session, through simple randomization and evenly distributed. During the familiarization session, information about the athletes was collected, including their communication characteristics as well as their knowledge of colors and letters. The three subsequent sessions were designated for the procedures of the C condition, LCE, and MF induction.

The test batteries were administered during the athletes' regular training hours, with a minimum interval of 24 h and a maximum of seven days between experimental conditions. Athletes were instructed to refrain from engaging in any type of physical exercise or sports training and were also advised to maintain regular sleep habits and to avoid consuming caffeine and similar meals within the 24 h preceding each test session.

The protocols for inducing low LCE, individualizing MF, and the C were conducted at the athletes' training sites. The C session involved no intervention. The LCE session consisted of watching a documentary for 30 min. For the individualization of MF, athletes were exposed to the Stroop task until they reached a minimum score of 6 on a 0–10 visual analog scale (VAS). Before initiating the testing battery (C, LCE, MF, sports performance, and physiological measures), the Perceived Recovery Status Scale (PRS) was administered.

The VAS was administered every 5 min and after the accuracy test during the LCE and MF experimental sessions. In the C session, it was collected before, after, and post-accuracy test. Heart rate variability was recorded in 5 min intervals during the LCE and MF interventions. The motivation was assessed before and after all three experimental conditions. Mental demand was measured after the LCE and MF interventions. Accuracy performance was recorded following the LCE and MF sessions, and after the administration of the PRS, VAS, and motivation questionnaires in the C session.

**Figure 1:**
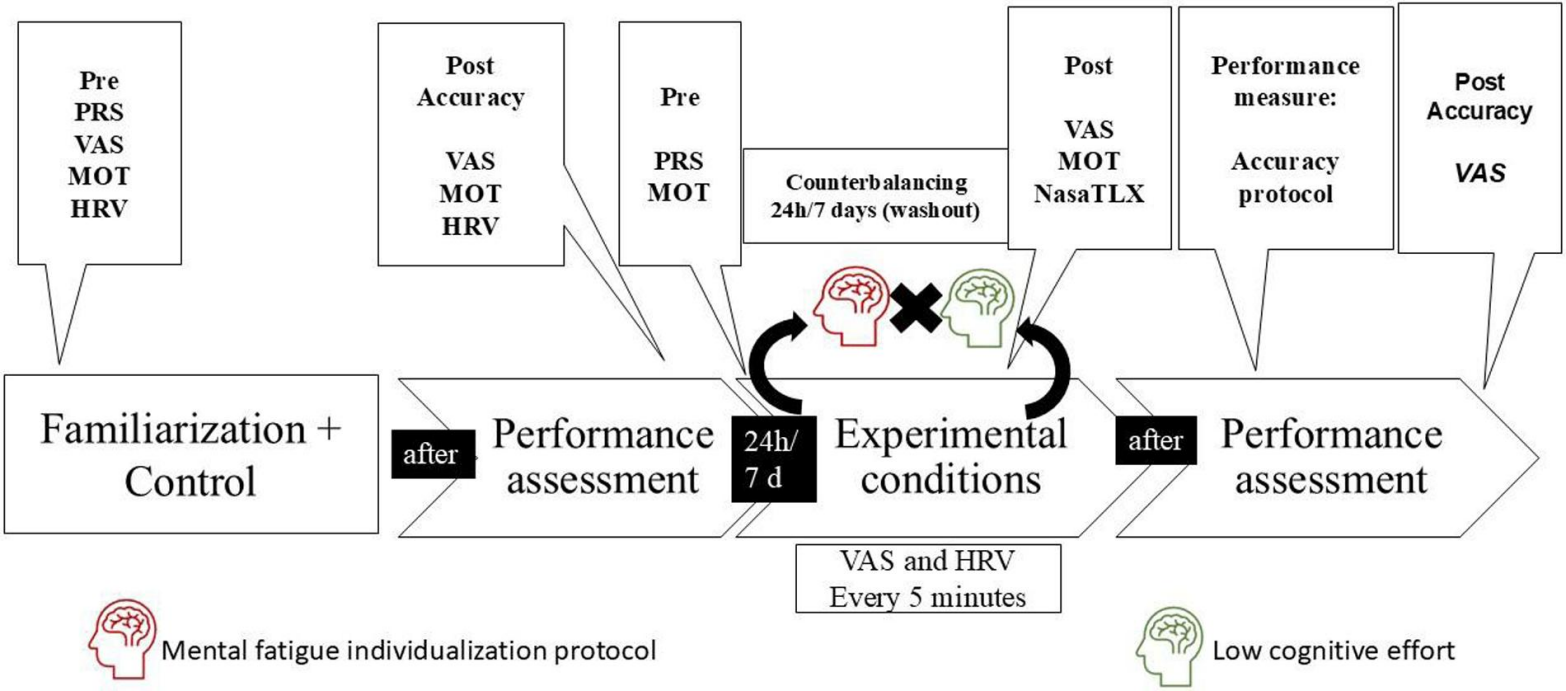
Data collection stages of the experimental protocol. MOT, motivation; VAS, visual analog scale; Pre, pre-conditions; Post, after the conditions; H, hours; D, days; HRV, heart rate variability.

All procedures are described in the following sections. An illustration of the experimental design is presented in Figure 1.

### 2.3 Protocol for mental fatigue individualization, low cognitive effort, and control condition

The MF was individualized using the printed Stroop Color Test, with participants continuing the task until reaching a score 6 on the VAS. The Stroop task requires cognitive inhibitory response and sustained attention (32). The task used in this investigation was the paper-based version employed in previous studies (8, 33).

Consisting of four words (red, blue, green, yellow), they are arranged in five columns on an A4 sheet, totaling 45 words per sheet, containing both congruent words and colors (where the spoken word matches the text color) and incongruent words and colors (where the spoken word differs from the text color). Participants have verbally responded to each word, with the correct response corresponding to the color of the word rather than the written text. For example, if the word “BLUE” is colored “GREEN,” the correct response should be “GREEN.”

Before starting the protocol, athletes with motor impairments were asked about their recognition of names and colors. Following procedures outlined in the PB arbitration manual, the researchers also ensured the use of athletes' specific gestures for communication, allowing for the use of auxiliary materials such as communication boards (vocabulary), computers with communication software, or even pointer devices like antenna pointers. The LCE condition consisted of watching a documentary video titled “Pantanal Matogrossense National Park” for a duration of 30 min. The C condition involved no interventions.

### 2.4 Measures

#### 2.4.1 Manipulation chek

##### 2.4.1.1 Heart rate variability assessment

Heart rate variability (HRV) was assessed with the participant seated in their own wheelchair, in a wakeful state, without sleeping, and advised to maintain a comfortable posture. The measurement was conducted using a heart rate monitor (Polar, RS 800 CX, United States of America) continuously for 5-min periods during the proposed interventions.

The analysis of autonomic cardiovascular modulation was conducted by measuring HRV during the proposed interventions (MF and LCE) and in the C group at the beginning and after the precision task, using spectral analysis techniques, in accordance with previous studies (34, 35).

The data related to the R-R interval (IP, ms) were obtained using a heart rate monitor (Polar, RS 800 CX, United States of America), which detects the electrocardiographic signal from one heartbeat to the next and transmits it to the pulse receiver, where the information is digitized, displayed, and stored.

Thus, the system detects ventricular depolarization corresponding to the R-wave of an electrocardiogram at a sampling frequency of 500 Hz and a temporal resolution of 1 ms². As a prerequisite, participants were required to have a regular night of sleep prior to the assessments and to consume light meals without caffeine.

For the analysis of the extracted data, the Kubios software (Kubios HRV Scientific, version 4.1.0) was used. The linear analysis method adopted was frequency domain analysis, evaluating low frequency (LF), high frequency (HF), and very low frequency (VLF) components using both Fast Fourier Transform (FFT) and autoregressive (AR) modeling approaches (AR) (36, 37). For the analysis, the first 5 min and the last 5 min of the LCE and MF interventions were considered, as well as the pre- and post-precision time points for the C group.

##### 2.4.1.2 Record of induced mental fatigue

To monitor the induction of MF, a 10-cm VAS was used, anchored with the expressions “low effort,” “moderate effort,” and “high effort.” The variables analyzed were “Perception of MF and LCE” (pre-induction, during the induction task, and post-precision), “C” (pre- and post-precision), and “Motivation” for the subsequent physical test (pre- and post-test).

For scale analysis, a ruler was used to measure the distance (in centimeters, to facilitate identification) between the starting point and the mark indicated by the researcher, based on the participant's verbal and/or gestural response. The values are reported as arbitrary units (a.u.). To facilitate signaling by the athlete, a central point was added to the analog scale to serve as a reference for verbal or gestural indication.

#### 2.4.2 Mental demand recording

To assess the mental demand induced by the LCE and MF conditions, the nasa-tlx was used, as adopted in previous studies (15). This is a 21-point anchored scale referring to the question: “*How mentally demanding was the previous task?*”.

#### 2.4.3 Throwing accuracy assessment

**Figure 2:**
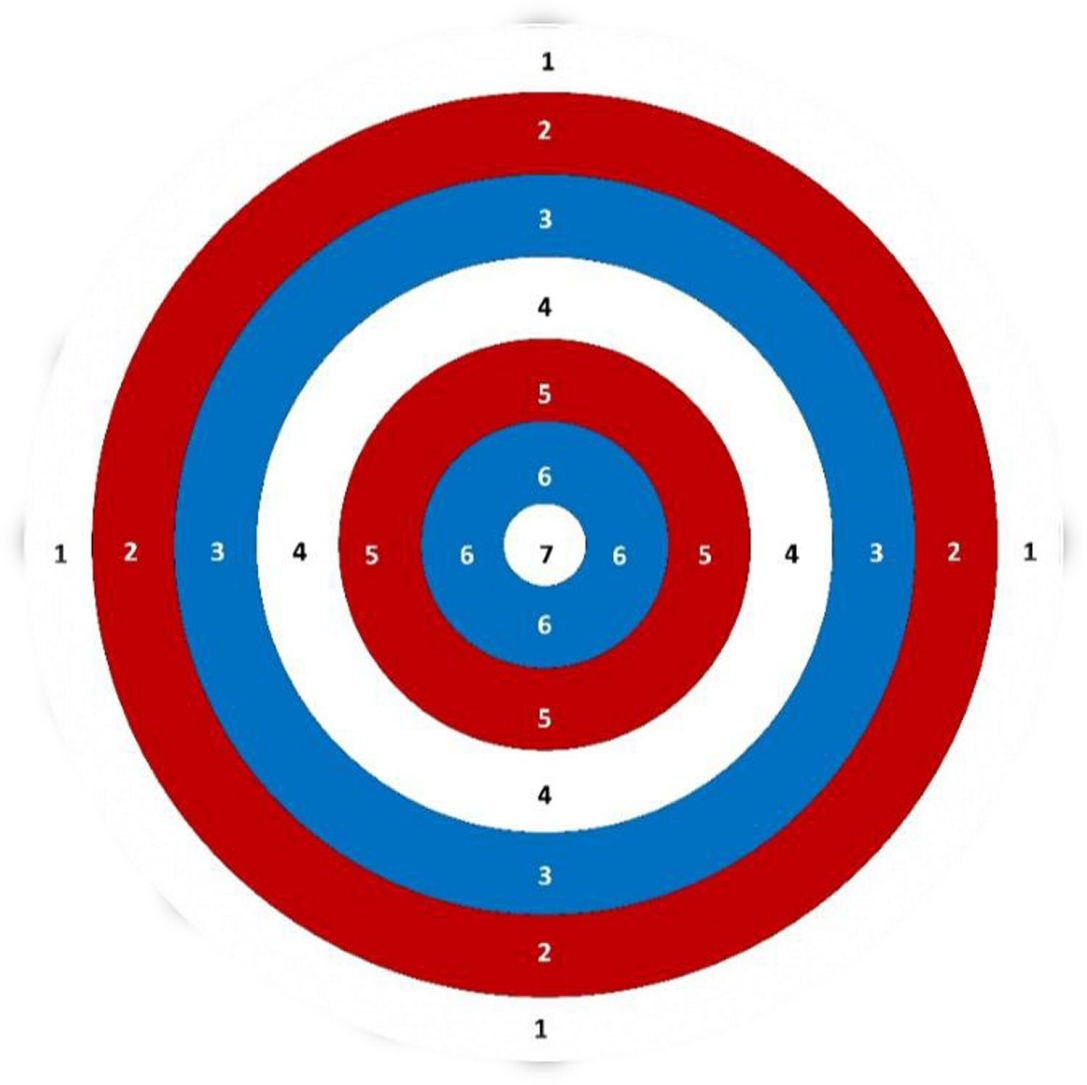
Target for assessing throwing performance in PB.

The accuracy assessment protocol for PB athletes has an intraclass correlation coefficient of 0.7 and a minimal detectable difference of 8 points (26, 38). Based on this, the present protocol consists of evaluating the throwing gesture specific to the sport, aimed at a target specially designed for this purpose (Figure 2) and positioned on the court floor. The target was developed using the dimensions of the athletes' own balls and has a diameter of 59.5 cm.

Were marked three distances on the court from the limit line of the boxes at 3, 6, and 9 m. The reference points used the center of the top lines of boxes number 2 and 5. Each player must position themselves in boxes 3 and 4 (in this order) and direct their throws laterally to the right when in box 4, and to the left when in box 3.

This positioning on the court was chosen to allow players an adjustment that is neither fully lateralized nor fully centered, ensuring an intermediate placement between attempts. Each player throws from both positions (right and left), totaling 12 throws (6 to the right and 6 to the left). Therefore, the maximum possible score for both actions (“right and left”) combined is 84.0 points; considering each action separately, the maximum possible score for each player is 42.0 points. Each player should select the balls for their throws, which may be specific balls from their own kits, for either the right or left side.

Before each throw, at each position on the court, the player may position their wheelchair in the direction of the throw without the time for each attempt being counted. Prior to the throws, a 2-min warm-up period must be given, using the balls chosen by the player. Each player has 30 s at each position to complete the throw in the designated spot. The 30-s time limit should be applied to all functional classes, encompassing the entire preparatory phase for the throws (e.g., rounding the balls, preparing the gutters, and “gutter breaks”).

At each position on the court, the player may perform two throws at the distances of 3, 6, and 9 m, both to the left and right, totaling 12 throws per athlete. To evaluate accuracy, it noted within which perimeter of the target the thrown ball stopped. The number corresponding to the perimeter must be recorded. If the athlete fails to reach the first perimeter of the target in either of the two throws, a value of 0.5 should be assigned for that throw.

In situations where the thrown ball lands between two perimeters, the larger portion (hemisphere) of the ball between the two perimeters should be considered. If it is not possible to determine which portion is larger between the two perimeters, the intermediate value between the two corresponding numbers should be assigned (e.g., if the thrown ball is between perimeters 3 and 5, a value of 4.0 should be assigned for that throw).

Similarly, when balls stop between two perimeters, the larger portion (hemisphere) of the ball between the two perimeters should be considered. If it is not possible to determine the larger portion, the value between the two achieved numbers should be assigned (e.g., if the white ball is between perimeters 3 and 4, a value of 3.5 should be assigned for that throw). In cases where both balls (the target ball and the opponent's ball) are positioned within the same perimeter, or for throws where the ball completely surpasses the target area, or even fails to reach the first established perimeter, a value of 0.5 is assigned.

The accuracy assessment protocol recommends using two targets; however, due to the extended time required to administer both targets, the attack target shown in Figure 2 was chosen. All athletes followed the same throwing order.

### 2.5 Statistical analysis

The assumptions of data normality were assessed using the Shapiro–Wilk test. Subsequently, a GEE model was employed to analyze the time effect pre, pos, and post-accuracy (post-a) for the C, LCE, and MF conditions, as well as for motivation and mental demand. The GEE was also used to evaluate the condition effect on accuracy at 3, 6, and 9 meters, and on total accuracy. The GEE model was selected based on data distribution, with a linear model adopted. Bonferroni *post hoc* tests were applied to identify mean differences among the analyzed data.

The effect size was calculated for all manipulation check using partial eta squared (*η_p_*^2^), which was employed to determine the main interaction effect, with the following interpretation thresholds: 0.10 (small), 0.30 (medium), and 0.50 (large) (39). All data were tabulated and analyzed using IBM SPSS Statistics 20.0 (IBM SPSS Statistics, Armonk, NY) and GraphPad Prism 8.0 (California Corporation®, USA). Results are presented as mean ± standard deviation for descriptive data and in median and range for the C, LCE, and MF data, and in median and confidence interval for the accuracy plots, with a significance level set at 5% (*p* < 0.05).

## 3 Results

Regarding the athletes, 54.5% were male and 45.5% were female. With respect to sport class, 27.3% were classified as BC1, 18.2% as BC2, 18.2% as BC3, and 36.4% as BC4. Concerning competitive levels, 9.1% were classified as level 2, 63.6% as level 3, 18.2% as level 4, and 9.1% as level 5. Additionally, 45.5% of the athletes were classified as having CP, while 54.5% were classified as non-CP, for further details, see Table 1.

**Table 1:** Descriptive information of the research participants.

ID	Age years	Sex	FC	AL	Type of disability	Exp. boccia, months	MF duration min	PRS C	PRS LEC	PRS MF
1	34	M	BC2	3	PC	96	30.88	7.5	5.0	7.5
2	39	M	BC4	3	NPC	132	11.57	6.0	6.0	8.0
3	23	F	BC2	2	PC	6	29.09	6.0	6.0	6.5
4	22	F	BC1	4	PC	84	70.05	6.5	6.5	6.5
5	35	M	BC4	3	NPC	120	30.26	6.0	6.5	6.0
6	18	M	BC3	3	NPC	60	19.06	6.0	6.0	6.0
7	26	F	BC4	4	NPC	72	14.22	5.5	6.0	5.5
8	24	F	BC1	5	PC	108	34.11	4.5	4.0	4.5
9	28	F	BC1	3	PC	132	10.77	6.0	4.5	6.0
10	54	M	BC4	3	NPC	108	36.91	5.5	5.5	5.5
11	26	M	BC3	3	PC	120	9.09	5.5	5.5	6.0
Mean	29.9	NA	NA	NA	NA	94.4	26.9	5.91	5.59	6.18
SD	10.1	NA	NA	NA	NA	37.6	17.6	0.7	0.8	0.9

ID, identifier; M, male; F, female; FC, functional class; AL, (athlete level) Type of disability; CP, cerebral palsy; NCP, non-cerebral palsy; Exp. Boccia, experience time with boccia; duration MF, stroop task response time; SD, standard deviation; C, control; LCE, low cognitive demand; MF, mental fatigue; PRS, perceived recovery status scale; NA, not applicable.

Table 1 presents the descriptive data of the 11 athletes who participated in all stages of the study.

### 3.1 Comparisons between manipulation checks

**Figure 3:**
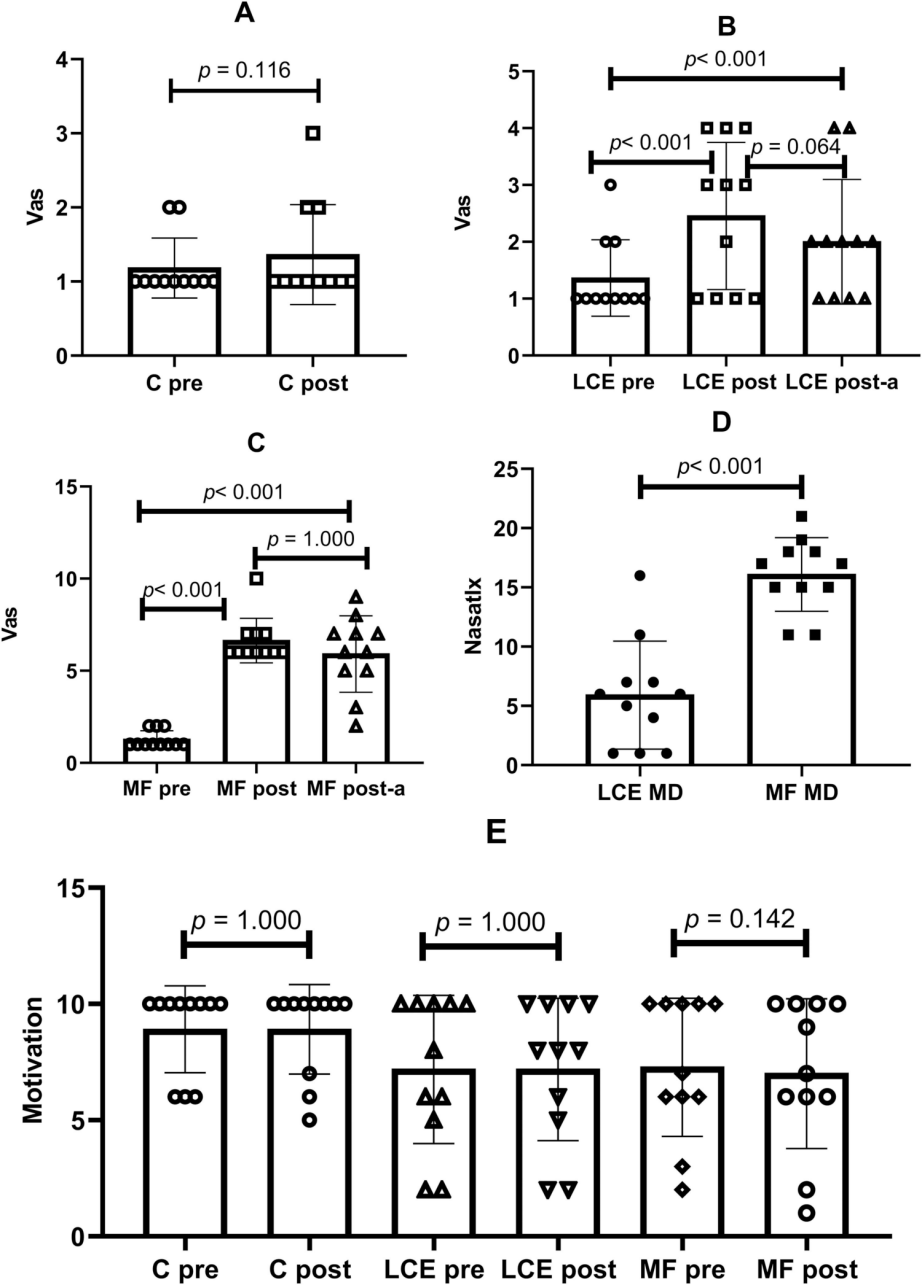
Comparisons between experimental conditions. C, control; LCE, low cognitive effort; MF, mental fatigue; VAS, visual analog scale; MD, mental demand; Pre, pre-conditions; Post, after the conditions; Post-a, after the accuracy test. **(A)** Pre and post comparison in the control condition C; **(B)** pre, post, and post-a comparisons in the LCE condition; **(C)** pre, post, and post-a comparisons in the MF condition; **(D)** comparison of MD between LCE and MF; **(E)** pre and post comparison of motivation in the C, LCE, and MF condition. The data are expressed as median and range.

The comparisons between the experimental conditions are presented in Figure 3.

Regarding the C condition, no significant time effects were observed (pre: 1.8 ± 0.11 vs. post: 1.38 ± 0.19; *p* = 0.118; *η_p_*^2^ = 0.182). In the LCE condition, significant differences over time were found (pre: 1.36 ± 0.19 vs. post: 2.45 ± 0.37; *p* < 0.001; *η_p_*^2^ = 0.595; pre: 1.36 ± 0.19 vs. post-a: 2.00 ± 0.31; *p* = 0.003; *η_p_*^2^ = 0.495), but no significant time effect was observed between post and post-A (post: 2.45 ± 0.37 vs. post-a: 2.00 ± 0.31; *p* = 0.064; *η_p_*^2^ = 0.325).

In the MF condition, there were significant time effects (pre: 1.27 ± 0.13 vs. post: 6.64 ± 0.34; *p* < 0.001; *η_p_*^2^ = 0.945; pre: 1.27 ± 0.13 vs. post-a: 5.91 ± 0.59; *p* < 0.001; *η_p_*^2^ = 0.879), with no significant time effect observed between post and post-a (post: 6.64 ± 0.34 vs. post-a: 5.91 ± 0.59; *p* = 1.000; *η_p_*^2^ = 0.075).

Regarding motivation, no significant time effects were observed for the C condition (pre: 8.91 ± 0.53 vs. post: 8.91 ± 0.55; *p* = 1.000; *η_p_*^2^ = 0.000), LCE condition (pre: 7.18 ± 0.91 vs. post: 7.18 ± 0.88; *p* = 1.000; *η_p_*^2^ = 0.000), or MF condition (pre: 7.27 ± 0.85 vs. post: 7.00 ± 0.92; *p* = 0.142; *η_p_*^2^ = 0.164). Additionally, a condition effect was observed for mental demand (LCE: 5.91 ± 1.3 vs. MF: 16.09 ± 0.89; *p* < 0.001; *η_p_*^2^ = 0.792), as illustrated in Figure 3.

Regarding HRV, no significant effect of time was found for any of the variables analyzed, as shown in Table 2.

**Table 2:** Comparison of HRV responses based on time.

Intervention	Variable	HRV	Pre	Post	*P*	*η_p_* ^2^
C	FFT	VLF	0.033 ± 0.001	0.033 ± 0.001	0.846	0.003
		LF	0.088 ± 0.007	0.090 ± 0.005	0.735	0.010
		HF	0.168 ± 0.006	0.201 ± 0.016	0.062	0.241
C	AR	VLF	0.036 ± 0.003	0.040 ± 0.000	0.294	0.091
		LF	0.101 ± 0.008	0.102 ± 0.006	0.911	0.001
		HF	0.150 ± 0.000	0.162 ± 0.011	0.294	0.091
LCE	FFT	VLF	0.031 ± 0.002	0.034 ± 0.001	0.755	0.101
		LF	0.080 ± 0.008	0.073 ± 0.009	1.000	0.049
		HF	0.216 ± 0.025	0.226 ± 0.026	1.000	0.007
LCE	AR	VLF	0.040 ± 0.000	0.036 ± 0.003	0.883	0.091
		LF	0.100 ± 0.005	0.092 ± 0.010	1.000	0.061
		HF	0.170 ± 0.019	0.181 ± 0.019	1.000	0.012
MF	FFT	VLF	0.033 ± 0.001	0.033 ± 0.001	1.000	0.002
		LF	0.078 ± 0.010	0.078 ± 0.007	1.000	0.000
		HF	0.180 ± 0.012	0.209 ± 0.020	0.400	0.170
MF	AR	VLF	0.033 ± 0.004	0.036 ± 0.003	0.883	0.091
		LF	0.120 ± 0.007	0.110 ± 0.008	1.000	0.063
		HF	0.162 ± 0.007	0.192 ± 0.027	0.675	0.118

HRV, heart rate variability; FFT, fast Fourier transform; AR, autoregressive model; VLF, low-frequency variability; LF, low frequency; HF, high frequency; C, control; LCE, low cognitive demand; MF, mental fatigue; *η_p_*^2^, effect size.

### 3.2 Comparisons of accuracy results

**Figure 4:**
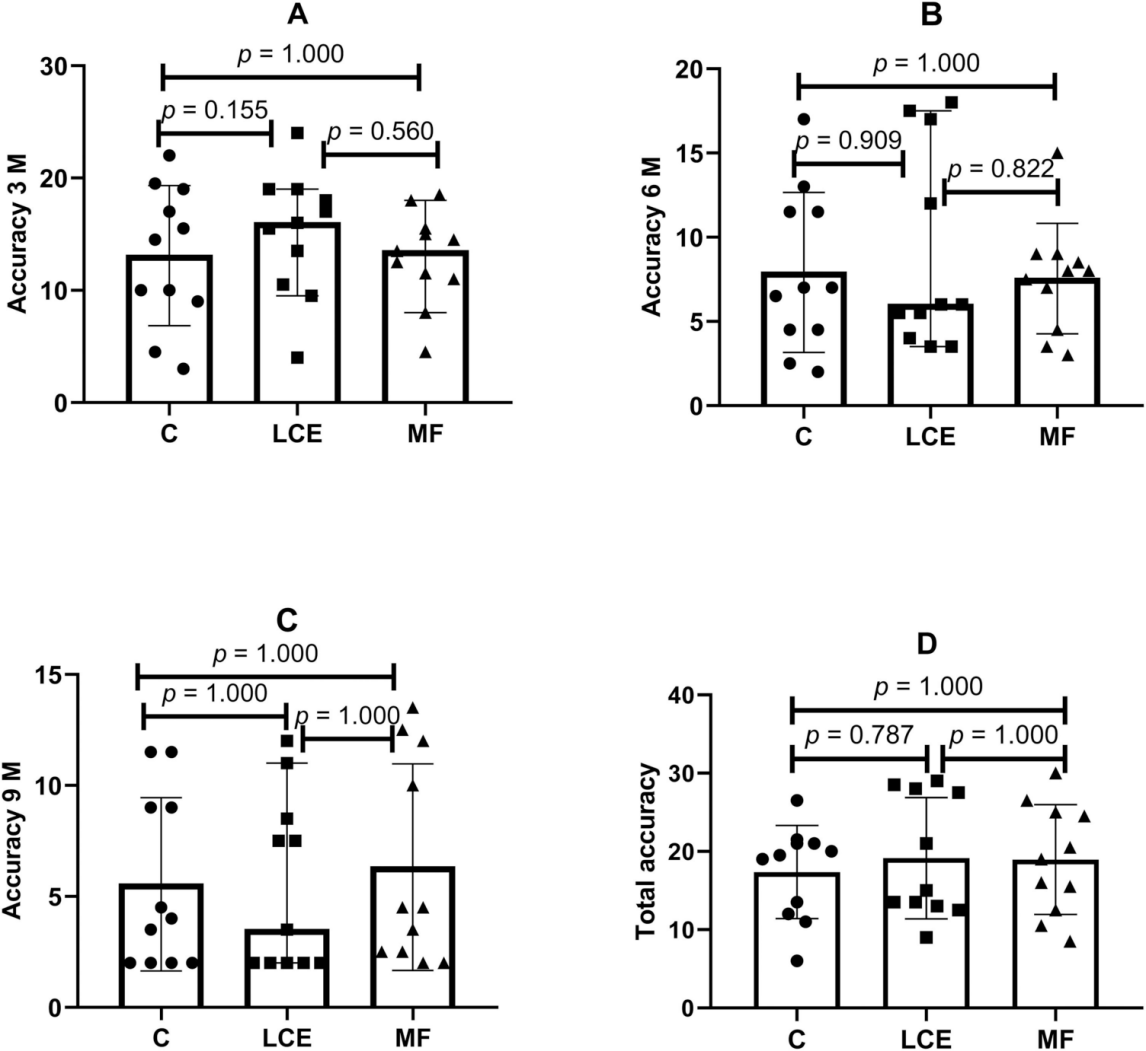
Comparison of accuracy results among the analyzed conditions. C, control; LCE, low cognitive demand; MF, mental fatigue; 3M, 3 meters; 6M, 6 meters; 9M, 9 meters. **(A)** Comparison of 3M accuracy across the C, LCE, and MF conditions; **(B)** comparison of 6M accuracy across the C, LCE, and MF conditions; **(C)** comparison of 9M accuracy across the C, LCE, and MF conditions; **(D)** comparison of total accuracy across the C, LCE, and MF conditions. The data are expressed as median and confidence interval.

Regarding accuracy, no significant effect of conditions was observed for the distances of 3 meters (C: 13.09 ± 1.7 vs. LCE: 15.09 ± 1.5; *p* = 0.155; *η_p_*^2^ = 0.256; C: 13.09 ± 1.7 vs. MF: 12.95 ± 1.1; *p* = 1.000; *η_p_*^2^ = 0.001; LCE: 15.09 ± 1.5 vs. MF: 12.95 ± 1.1; *p* = 0.560; *η_p_*^2^ = 0.137), 6 meters (C: 7.90 ± 1.3 vs. LCE: 8.95 ± 1.7; *p* = 0.909; *η_p_*^2^ = 0.088; C: 7.90 ± 1.3 vs. MF: 7.5 ± 0.9; *p* = 1.000; *η_p_*^2^ = 0.012; LCE: 8.95 ± 1.7 vs. MF: 7.5 ± 0.9; *p* = 0.822; *η_p_*^2^ = 0.098), 9 meters (C: 5.54 ± 1.1 vs. LCE: 5.4 ± 1.1; *p* = 1.000; *η_p_*^2^ = 0.000; C: 5.54 ± 1.1 vs. MF: 6.3 ± 1.3; *p* = 1.000; *η_p_*^2^ = 0.027; LCE: 5.4 ± 1.1 vs. MF: 6.3 ± 1.3; *p* = 1.000; *η_p_*^2^ = 0.031), and total accuracy (C: 17.3 ± 1.7 vs. LCE: 19.1 ± 2.2; *p* = 0.787; *η_p_*^2^ = 0.103; C: 17.3 ± 1.7 vs. MF: 18.9 ± 2.0; *p* = 1.000; *η_p_*^2^ = 0.064; LCE: 19.1 ± 2.2 vs. MF: 18.9 ± 2.0; *p* = 1.000; *η_p_*^2^ = 0.001). As shown in Figure 4.

## 4 Discussion

The primary objectives of the present study were: (1) to verify the effectiveness of the experimental conditions MF and LCE in inducing MF in the sample; and (2) to examine the influence of MF and LCE on HRV, motivation, and accuracy in boccia athletes.

Based on these findings, the results of the present study indicate significant time effects for the LCE and MF conditions, demonstrating that both conditions were effective in inducing MF in the athletes. No significant time effects were observed between the post and post-precision moments for LCE and MF, which supports the persistence of a state of heightened cognitive effort following the precision test. Furthermore, no significant time effect was found in the C group. Additionally, a condition effect was observed for MF, which induced greater cognitive load compared to LCE, as measured by the nasa-tlx. According to the analyses conducted for HRV and motivation, no significant time effects were detected. Regarding the accuracy analyses, no condition effects were observed for the distances of 3, 6, and 9 m, nor for total accuracy.

Based on the above, the present study represents an innovative contribution to the field of sports science by proposing the individualization of MF in a Paralympic sport for which no prior research has documented this phenomenon in boccia athletes. As such, this is the first study to investigate the influence of individualized MF on the technical and physiological capacities of PB athletes. Accordingly, the results related to the induction of MF proved to be successful. These findings are consistent with previous studies that also demonstrated the induction of MF through the Stroop task in both athletes and para-athletes (16, 40–43).

Moreover, the average time for MF induction was 26.9 min, whereas the literature suggests that stimuli intended to induce MF should last ≥30 min (2, 44), However, this shorter MF induction time in PB athletes may be justified by the fact that individuals with CP are more susceptible to MF than the general population (22, 24), Thus, this highlights the need for individualizing MF in PB athletes, considering that a recent study demonstrated an average induction time of 25 min to elicit MF in wheelchair basketball athletes (16). However, it is possible to observe an effect within 5 min of a cognitive task to induce MF, as demonstrated in a previous study (45).

At the same time, the present study also demonstrated that LCE demands are capable of significantly increasing mental effort in PB athletes. These findings contrast with those reported in the literature, which indicate that activities involving videos do not significantly increase mental demand (46), However, the increase in mental effort may occur over time in the group that watched the documentary, which is consistent with the findings of the present study (43). Furthermore, the MF group exhibited a higher cognitive load than the LCE group. Similarly, a previous study observed that the MF condition induced a greater mental load compared to the control group, which involved watching a documentary (15).

Regarding HRV, no significant differences were found in any of the analyses conducted in the present study, which contrasts with the findings of previous studies (15), which found lower HRV for the MF condition compared to the C. Furthermore, very low frequency (VLF) increases over time, and low frequency (LF) increases during the Stroop task compared to the control group (47), which was not observed in the present study. Furthermore, a systematic review found that HRV is considered a reliable indicator of MF, as HRV undergoes changes based on the duration of tasks that induce MF, indicating that HRV is an autonomic marker for MF (48). These changes, however, were not observed in the present study. However, such observations may not be confirmed due to the use of pre- and post-comparisons instead of analyses conducted during the cognitive effort process, as well as limitations related to sample size or interindividual variability.

Thus, motivation also did not show significant differences over time, corroborating the results of previous studies conducted in football, handball, and badminton and wheelchair basketball (16, 43, 46, 49, 50). Such results may be justified by the fact that this is a routine task in PB, or by the lack of a sport-specific questionnaire to assess motivation.

Among the analyses related to accuracy, no significant differences were observed. This finding aligns with previous evidence showing that MF does not affect the accuracy and aiming performance of soldiers (15). However, numerous findings in the literature demonstrate that MF decreases accuracy in sports (8, 14, 40, 41, 43, 51, 52). However, it is possible that the sample size was insufficient to detect effects on athletes' accuracy. Another possibility is that the accuracy test was not sensitive enough to capture differences, given that the minimum detectable difference of the test is 8 points (26, 38). Another point is that, instead of applying both targets attack and defense only the attack target was applied, which may have influenced the accuracy results.

From a practical standpoint, the results of the present study indicate that boccia athletes are more susceptible to both low and high cognitive demands, which can lead to states of MF. Furthermore, it is not clear whether MF or LCE negatively affects accuracy in PB athletes.

## 5 Study limitations

Among the limitations of the present study are the heterogeneity and sample size. Furthermore, the paper-based Stroop task presents limitations beyond response measurement, such as a lack of ecological validity compared to more context-specific Stroop tasks for boccia using computers or smartphones. Additionally, the absence of kinematic analyses of boccia throws to assess possible strategies employed to maintain accuracy, as well as the lack of randomization of the throwing order in the precision test, are also noted limitations. Which may allow for adaptability in the execution of the test. Given that compensations in kinematics or adaptability to the throwing order may have occurred, thereby minimizing the reduction in accuracy.

## 6 Strengths

As strengths of the present study, the high external validity stands out, in addition to being the first intervention study on MF) and PB in the field of sports sciences. Finally, it is notable for employing a performance assessment tool specifically designed for the analyzed sport modality.

## 7 Conclusion

The present study found that the individualization process of MF was successful, and that the LCE activity also increases the perception of mental effort in PB athletes. Another finding was that the MF condition elicited higher levels of cognitive load compared to LCE. No evidence was found to suggest that MF or LCE impair accuracy under current conditions in the PB athletes.

Thus, the experimental conditions did not show significant differences regarding HRV and motivation. These results indicate that athletes of different competitive levels in PB are susceptible to both low and high cognitive efforts. Therefore, coaches and athletes should implement routines to monitor cognitive effort, especially during competitive periods.

The present study demonstrated that the individualization of MF in PB athletes is an effective approach to induce MF in this population, and that the use of specific protocols to assess the technical capacity of PB is necessary in future research. Finally, further studies are needed to better understand the effects of LCE and MF on the performance of PB athletes.

## Data Availability

The original contributions presented in the study are included in the article/Supplementary Material, further inquiries can be directed to the corresponding authors.
